# Attachment insecurity, professional quality of life, and perceived posttraumatic growth among hospital nurses

**DOI:** 10.3389/fpsyg.2026.1931294

**Published:** 2026-09-23

**Authors:** Barbara Colombo, Joyce Berhold, Chiara Fioretti

**Affiliations:** 1 Affect and Adaptation (A^2^) Lab, School of Psychology, Fielding Graduate University, Santa Barbara, CA, United States; 2 Department of Human, Philosophical and Educational Sciences, University of Salerno, Salerno, Italy

**Keywords:** attachment insecurity, burnout, compassion satisfaction, posttraumatic growth, professional quality of life, secondary traumatic stress

## Abstract

**Background:**

Hospital nurses routinely manage emotionally demanding clinical work that may contribute to burnout and secondary traumatic stress, while also providing opportunities for meaning making processes, compassion satisfaction, and perceived posttraumatic growth. Attachment insecurity may shape how nurses regulate distress and engage with supportive relationships, but its associations with both negative and positive professional outcomes remain understudied.

**Objective:**

This study examined associations among attachment anxiety, attachment avoidance, professional quality of life, and perceived posttraumatic growth among hospital nurses providing direct patient care.

**Methods:**

A cross-sectional online survey was completed by 208 hospital-based registered nurses and licensed practical nurses in the United States. Participants completed the Experiences in Close Relationships–Short Form, Professional Quality of Life Scale, and Posttraumatic Growth Inventory. Hierarchical multiple regression analyses examined associations of attachment anxiety and avoidance with compassion satisfaction, burnout, secondary traumatic stress, total perceived posttraumatic growth, and growth in relating to others. Age was included as a covariate in all models; high-intensity clinical setting was additionally included in the burnout and secondary traumatic stress models.

**Results:**

Attachment avoidance was consistently associated with less favorable outcomes. Greater avoidance was associated with lower compassion satisfaction (*β* = −0.19, *p* = 0.006), greater burnout (*β* = 0.40, *p* < 0.001), greater secondary traumatic stress (*β* = 0.45, *p* < 0.001), lower perceived relational growth (*β* = −0.15, *p* = 0.012), and lower total perceived posttraumatic growth (*β* = −0.15, *p* = 0.013). Attachment anxiety was independently associated with greater burnout (*β* = 0.31, *p* < 0.001) and secondary traumatic stress (*β* = 0.33, *p* < 0.001), but was not uniquely associated with compassion satisfaction or perceived posttraumatic growth. Compassion satisfaction was positively associated with relational growth (*β* = 0.34, *p* < 0.001) and total perceived posttraumatic growth (*β* = 0.36, *p* < 0.001). The final models explained 31.8% of the variance in relational growth, 30.0% in total perceived posttraumatic growth, 39.6% in burnout, and 49.8% in secondary traumatic stress. High-intensity clinical setting was associated with greater burnout and secondary traumatic stress.

**Conclusion:**

Attachment insecurity, particularly attachment avoidance, was associated with both negative and positive dimensions of nurses’ professional experiences. Compassion satisfaction was associated with greater perceived growth. Findings identify emotional regulation, supportive connection, professional meaning, and organizational working conditions as potentially relevant targets for future resilience-oriented intervention research. Cross-sectional self-report data preclude causal conclusions, and the posttraumatic growth measure was not anchored to nursing-related events.

## 1 Introduction

Nursing involves sustained exposure to emotionally demanding clinical situations, including patient suffering, uncertainty, acute illness, and death. These demands often occur alongside high workloads, time pressure, and ongoing emotional labor, placing nurses at risk for burnout and secondary traumatic stress (Bahari et al., 2022; Jarrad and Hammad, 2020; Yılmaz and Üstün, 2018). At the same time, emotionally demanding work does not lead uniformly to distress (Mopkins, 2022). Many nurses also experience fulfillment, meaning, and satisfaction through their professional roles (Guastello et al., 2024). Understanding both the harmful and potentially sustaining aspects of caregiving work is therefore important for supporting nurses’ wellbeing.

Professional quality of life provides a useful framework for examining these divergent experiences. The model includes compassion satisfaction, burnout, and secondary traumatic stress. Compassion satisfaction refers to the fulfillment derived from helping others and making a meaningful contribution through one’s work, whereas burnout and secondary traumatic stress reflect negative occupational consequences of prolonged caregiving demands (Bahari et al., 2022; Yılmaz and Üstün, 2018). Research on nurses’ well-being has often focused primarily on burnout and compassion fatigue; however, a more complete account should also consider the psychological factors that may shape both distress and positive professional meaning (Sinclair et al., 2017; Zhang et al., 2018).

Attachment theory offers one additional framework for understanding why nurses may differ in their responses to emotionally demanding work. Adult attachment insecurity is commonly conceptualized along two dimensions: attachment anxiety and attachment avoidance (Eilert and Buchheim, 2023; Mikulincer and Shaver, 2019; Stein et al., 2002). Attachment anxiety is characterized by heightened sensitivity to rejection, fear of abandonment, and increased reassurance seeking. In contrast, attachment avoidance is characterized by discomfort with closeness, emotional distancing, and a tendency to suppress dependency needs (Eilert and Buchheim, 2023; Mikulincer and Shaver, 2019; Simpson and Rholes, 2017). These dimensions are relevant to nursing because they are associated with differences in emotion regulation, support seeking, interpersonal functioning, and responses to stress. Among the healthcare professions, nursing is distinguished by the provision of continuous care relationships, which are often shaped by patients’ dependence on healthcare professionals to meet their clinical, organizational, and emotional needs (Piredda et al., 2015).

In healthcare and other helping contexts, attachment-related emotion-regulation strategies may be particularly important. Nurses must often remain emotionally responsive to patients and colleagues while managing repeated exposure to distressing situations (Salmond et al., 2019). Hyperactivating strategies associated with attachment anxiety may contribute to heightened emotional distress and difficulty disengaging from stressful experiences (Eilert and Buchheim, 2023; Missouridou, 2017). Conversely, deactivating strategies associated with attachment avoidance may reduce willingness to seek support, engage emotionally, or derive relational reward from caregiving interactions (Kelly, 2020; Mikulincer and Shaver, 2019). Previous work suggests that attachment insecurity is associated with emotional exhaustion and caregiving-related difficulties among nurses, supporting the relevance of attachment processes to professional quality of life (Golia et al., 2017; Ren et al., 2024).

Attachment may also be relevant to perceived posttraumatic growth. Posttraumatic growth refers to perceived positive psychological changes that may emerge through efforts to cope with highly challenging experiences; it does not imply the absence of distress (Tedeschi and Calhoun, 2004). Such growth may include changes in personal strength, appreciation of life, new possibilities, spiritual growth, and relationships with others (Tedeschi and Calhoun, 1996). The Relating to Others domain may be particularly aligned attachment because it involves greater emotional closeness, openness, appreciation of support, and investment in interpersonal relationships. Although the present study did not assess growth specifically attributable to nursing experiences, perceived posttraumatic growth reported by nurses may be associated with the ways in which individuals process, regulate, and make meaning of challenging experiences (Wang et al., 2024). A substantial body of literature has documented the occurrence of secondary post-traumatic growth among healthcare professionals who provide daily care to individuals experiencing severe illness. This evidence suggests that exposure to patients’ traumatic experiences may foster vicarious experiences of personal growth among healthcare providers (Cohen and Collens, 2013).

Prior evidence suggests that attachment insecurity may be relevant to posttraumatic growth, although associations appear to differ across attachment dimensions and contexts (Arikan et al., 2016; Gleeson et al., 2021). Attachment avoidance may be particularly likely to interfere with relational growth because distancing strategies can limit emotional engagement and reliance on supportive relationships. Evidence concerning attachment anxiety and posttraumatic growth is less consistent, potentially because heightened emotional engagement may be associated with both distress and efforts to make meaning of difficult experiences (Gleeson et al., 2021; Huh et al., 2020). Accordingly, attachment avoidance was expected to show a clearer negative association with perceived growth than attachment anxiety.

Compassion satisfaction may represent an additional professional resource associated with perceived growth. Nurses who experience their work as meaningful and rewarding may be more likely to maintain a sense of purpose, connection, and engagement despite occupational stress (Zeng et al., 2023). Compassion satisfaction may therefore be positively associated with perceived posttraumatic growth, particularly growth in relationships. Examining compassion satisfaction alongside attachment insecurity may help clarify whether positive professional meaning is associated with perceived growth beyond individual differences in relational insecurity.

Despite these conceptual links, research has rarely examined attachment anxiety, attachment avoidance, compassion satisfaction, burnout, secondary traumatic stress, and perceived posttraumatic growth within a single framework among hospital-based nurses. Much of the existing literature has focused on occupational distress, with comparatively less attention to positive professional experiences and perceived growth.

The present cross-sectional study examined associations among attachment anxiety, attachment avoidance, professional quality of life, and perceived posttraumatic growth among hospital-based nurses providing direct patient care. First, the study examined whether attachment anxiety and avoidance were associated with compassion satisfaction, burnout, and secondary traumatic stress. It was expected that greater attachment anxiety and avoidance would be associated with greater burnout and secondary traumatic stress, whereas greater attachment avoidance would be associated with lower compassion satisfaction. Second, the study examined whether attachment insecurity and compassion satisfaction were associated with overall perceived posttraumatic growth and growth in relating to others. Greater attachment avoidance was expected to be associated with lower perceived growth, whereas greater compassion satisfaction was expected to be associated with greater perceived growth. Given mixed prior findings, associations between attachment anxiety and posttraumatic growth were examined exploratorily.

## 2 Methods

### 2.1 Participants

Eligible participants were registered nurses or licensed practical nurses who were at least 21 years old, currently worked in hospital-based settings, and provided direct patient care. Nurses working exclusively in outpatient or administrative roles and those not currently employed in hospital settings were excluded.

The final sample comprised 208 nurses currently working in hospital settings and providing direct patient care in the United States. Participants ranged in age from 23 to 75 years (*M* = 40.37, *SD* = 12.48) and reported between 2 and 40 years of nursing experience (*M* = 12.54, *SD* = 9.00).

Most participants identified as female (*n* = 167, 80.3%), while 41 participants identified as male (19.7%). Regarding racial/ethnic identity, 117 participants identified as White/Caucasian (56.3%), 40 as Hispanic or Latino (19.2%), 30 as Black or African American (14.4%), 17 as Asian (8.2%), and 4 as American Indian or Alaska Native (1.9%).

### 2.2 Procedure

This study used an anonymous, cross-sectional online survey design and was conducted entirely online using Qualtrics. Participants were recruited through hospital networks with administrative permission. Recruitment involved 23 hospitals across multiple U. S. states, including Vermont, New York, New Hampshire, Florida, North Carolina, South Carolina, Oregon, Connecticut, Massachusetts, and California. Participating institutions included a mix of academic, private, and public hospitals. Recruitment materials were disseminated through a combination of hospital email lists, internal newsletters or communication platforms, flyers, and distribution through hospital administrators or managers. Because recruitment relied on anonymous survey links distributed through multiple institutional channels, the number of nurses who received or viewed the invitation could not be determined, and a formal response rate could not be calculated. Twelve incomplete survey responses were excluded prior to analysis. Participation was voluntary, and informed consent was obtained electronically before any data were collected. A waiver of signed consent was requested due to the anonymous design of the study. The study was approved by the Institutional Review Board of Fielding Graduate University (Protocol No. IRB 25–060) and was conducted in accordance with the Declaration of Helsinki. Total participation time was estimated to be approximately 20 to 30 min.

After providing consent, participants completed a demographic questionnaire assessing age, gender, racial/ethnic identity, years of nursing experience, and clinical practice area. Participants then completed self-report measures of attachment, professional quality of life, and perceived posttraumatic growth. Participants were allowed to skip any question and could exit the survey at any time without penalty. At the end of the survey, they were provided with general mental health resources in case participation elicited distress.

Data quality was evaluated prior to analysis. Incomplete survey responses were excluded, and responses were screened for duplicate submissions and implausibly short completion times. As an additional indicator of participant engagement, responses to an open-ended narrative task included at the end of the survey were reviewed for response length, coherence, and logical relevance to the prompt. Although responses to this narrative task were not included in the analyses reported in the present study, they provided an additional check on meaningful engagement with the survey. These procedures were used to evaluate the quality and completeness of the responses retained for analysis.

### 2.3 Measures

Experiences in Close Relationships–Short Form (ECR-S) (Wei et al., 2007). Adult attachment was assessed using the Experiences in Close Relationships–Short Form (ECR-S), a 12-item self-report measure of attachment anxiety and attachment avoidance. Items are rated on a 1 to 7 scale, with higher scores indicating greater attachment anxiety or avoidance. Mean subscale scores were calculated. In the current sample, internal consistency was good for both attachment anxiety (α = 0.82) and attachment avoidance (α = 0.88).

Professional Quality of Life Scale (ProQOL) (Stamm, 2009). Professional quality of life was assessed using the Professional Quality of Life Scale, which includes three 10-item subscales: Compassion Satisfaction, Burnout, and Secondary Traumatic Stress. Items are rated on a 5-point frequency scale, with higher scores indicating greater levels of each construct. Internal consistency in the current sample was good to excellent for Compassion Satisfaction (*α* = 0.88), Burnout (*α* = 0.82), and Secondary Traumatic Stress (*α* = 0.89).

Posttraumatic Growth Inventory (PTGI) (Tedeschi and Calhoun, 1996). Perceived posttraumatic growth was assessed using the 21-item Posttraumatic Growth Inventory. Items are rated on a 6-point scale ranging from 0 (*I did not experience this change as a result of my crisis*) to 5 (*I experienced this change to a very great degree*). The PTGI yields a total score and subscale scores, including Relating to Others. Internal consistency was excellent for the total score (*α* = 0.95) and for the Relating to Others subscale (*α* = 0.92). Participants were not instructed to anchor their PTGI responses to a specific nursing-related event or occupational experience; accordingly, scores are interpreted as perceived posttraumatic growth reported by nurses rather than growth attributable specifically to nursing work.

### 2.4 Analytic strategy

All analyses were run using IBM SPSS version 31.

Descriptive statistics and Pearson correlations were calculated for demographic variables and the primary study measures. Because age and years of nursing experience were strongly correlated (*r* = 0.79, *p* < 0.001), age was retained as the primary demographic covariate in the posttraumatic growth models. This choice was based both on its stronger bivariate associations with the PTGI outcomes and on conceptual considerations. Because the PTGI was not anchored specifically to occupational experiences, age was considered a broader indicator of developmental and cumulative life-experience differences that may be relevant to perceived posttraumatic growth, whereas years of nursing experience primarily reflects occupational tenure.

Hierarchical multiple regression analyses were conducted to examine associations among attachment insecurity, professional quality of life, and perceived posttraumatic growth. For models predicting the PTGI Relating to Others subscale and PTGI total score, age was entered in Step 1, attachment anxiety and attachment avoidance were entered in Step 2, and compassion satisfaction was entered in Step 3. For the compassion satisfaction model, age was entered in Step 1 and attachment anxiety and avoidance were entered in Step 2. For burnout and secondary traumatic stress, age and high-intensity clinical setting were entered in Step 1, followed by attachment anxiety and attachment avoidance in Step 2. High-intensity clinical setting was coded as 0 = no and 1 = yes, based on whether participants reported working in at least one high-intensity clinical setting. Intensive care, emergency/trauma, oncology, and infectious disease units were categorized as high-intensity settings. Regression assumptions and influential cases were examined using residual diagnostics, variance inflation factors, Mahalanobis distance, and Cook’s distance.

## 3 Results

### 3.1 Preliminary analyses

Following the data-quality screening procedures described above, all analyses included the final sample of 208 participants. There were no missing values on the variables included in the present analyses. Descriptive statistics and bivariate correlations among the primary study variables are presented in Table 1. Age and years of nursing experience were strongly correlated, *r* = 0.79, *p* < 0.001. Because age showed the stronger bivariate associations with both posttraumatic growth outcomes, age was retained as the demographic covariate in the primary posttraumatic growth models.

**Table 1:** Descriptive statistics and bivariate correlations among primary study variables.

Variable	*M*	SD	1	2	3	4	5	6	7	8	9
1. Age	40.37	12.48									
2. Years of nursing experience	12.54	9.00	0.785**								
3. Attachment anxiety	3.79	1.24	−0.229**	−0.204**							
4. Attachment avoidance	2.74	1.16	−0.006	0.228**	0.132						
5. Compassion satisfaction	39.49	5.41	0.123	0.131	−0.026	−0.188**					
6. Burnout	24.25	6.10	−0.068	−0.129	0.422**	0.474**	−0.611**				
7. Secondary traumatic stress	24.85	7.70	−0.279**	−0.186**	0.480**	0.520**	−0.253**	0.704**			
8. PTGI total	64.75	21.85	−0.347**	−0.216**	0.162*	−0.204**	0.342**	−0.389**	0.094		
9. PTGI Relating to Others	20.82	8.79	−0.400**	−0.246**	0.158*	−0.200**	0.311**	−0.393**	0.081	0.926**	

*N* = 208. Pearson correlations are reported. PTGI = Posttraumatic Growth Inventory. *p < 0.05. **p < 0.01.

At the bivariate level, attachment avoidance was negatively associated with compassion satisfaction, total posttraumatic growth, and growth in relating to others, and was positively associated with burnout and secondary traumatic stress. Attachment anxiety was positively associated with burnout, secondary traumatic stress, total posttraumatic growth, and growth in relating to others. Compassion satisfaction was positively associated with both total posttraumatic growth and growth in relating to others, whereas burnout was negatively associated with both posttraumatic growth outcomes.

### 3.2 Attachment, compassion satisfaction, and posttraumatic growth

Hierarchical regression results predicting relational growth and total posttraumatic growth are summarized in Table 2. A hierarchical multiple regression examined associations of age, attachment anxiety, attachment avoidance, and compassion satisfaction with the PTGI Relating to others subscale. Age was entered in the first step, attachment anxiety and avoidance were entered in the second step, and compassion satisfaction was entered in the final step.

**Table 2:** Hierarchical regression analyses predicting relational and total posttraumatic growth.

Predictor	PTGI relating to others *B*	SE *B*	*β*	95% CI for *B*	*p*	PTGI Total *B*	SE *B*	*β*	95% CI for *B*	*p*
Age	−0.297	0.042	−0.422	[−0.381, −0.214]	< 0.001	−0.645	0.107	−0.368	[−0.855, −0.435]	< 0.001
Attachment anxiety	0.641	0.428	0.090	[−0.202, 1.484]	0.135	1.899	1.077	0.107	[−0.224, 4.022]	0.079
Attachment avoidance	−1.144	0.452	−0.151	[−2.035, −0.253]	0.012	−2.864	1.138	−0.152	[−5.107, −0.620]	0.013
Compassion satisfaction	0.548	0.097	0.337	[0.357, 0.739]	< 0.001	1.463	0.244	0.362	[0.982, 1.943]	< 0.001
Model fit										
Block 1 *R*^2^	0.160					0.121				
Block 2 Δ*R*^2^	0.050					0.055				
Block 3 Δ*R*^2^	0.108					0.124				
Final *R*^2^	0.318					0.300				
Adjusted *R*^2^	0.304					0.286				
Final *F*(df)	23.65 (4, 203), *p* < 0.001					21.76 (4, 203), *p* < 0.001				

Unstandardized coefficients are reported with 95% confidence intervals are reported. Block 1 included age; Block 2 added attachment anxiety and attachment avoidance; Block 3 added compassion satisfaction. PTGI = Posttraumatic Growth Inventory.

Age explained 16.0% of the variance in relational growth, *R*^2^ = 0.160, *F*(1, 206) = 39.25, *p* < 0.001. Adding attachment anxiety and avoidance explained an additional 5.0% of variance, Δ*R*^2^ = 0.050, Δ*F*(2, 204) = 6.48, *p* = 0.002. Compassion satisfaction explained a further 10.8% of variance, Δ*R*^2^ = 0.108, Δ*F*(1, 203) = 32.05, *p* < 0.001. The final model was significant, *R*^2^ = 0.318, adjusted *R*^2^ = 0.304, *F*(4, 203) = 23.65, *p* < 0.001.

In the final model, younger age was associated with greater relational growth, *B* = −0.30, *β* = −0.42, *p* < 0.001. Higher attachment avoidance was associated with lower relational growth, *B* = −1.14, *β* = −0.15, *p* = 0.012, whereas greater compassion satisfaction was associated with greater relational growth, *B* = 0.55, *β* = 0.34, *p* < 0.001. Attachment anxiety was not uniquely associated with relational growth after the other variables were considered, *B* = 0.64, *β* = 0.09, *p* = 0.135.

A secondary hierarchical regression was conducted using total PTGI score as the outcome. Age explained 12.1% of the variance in total posttraumatic growth, *R*^2^ = 0.121, *F*(1, 206) = 28.26, *p* < 0.001. Attachment anxiety and avoidance explained an additional 5.5% of variance, Δ*R*^2^ = 0.055, Δ*F*(2, 204) = 6.84, *p* = 0.001, and compassion satisfaction explained a further 12.4% of variance, Δ*R*^2^ = 0.124, Δ*F*(1, 203) = 36.04, *p* < 0.001. The final model explained 30.0% of the variance in total posttraumatic growth, adjusted *R*^2^ = 0.286, *F*(4, 203) = 21.76, *p* < 0.001.

Younger age, lower attachment avoidance, and greater compassion satisfaction were independently associated with greater total posttraumatic growth. Specifically, age was negatively associated with PTGI total score, *B* = −0.65, *β* = −0.37, *p* < 0.001; attachment avoidance was negatively associated with PTGI total score, *B* = −2.86, *β* = −0.15, *p* = 0.013; and compassion satisfaction was positively associated with PTGI total score, *B* = 1.46, *β* = 0.36, *p* < 0.001. Attachment anxiety was not uniquely associated with total posttraumatic growth, *B* = 1.90, *β* = 0.11, *p* = 0.079.

As a sensitivity analysis, a model including both age and years of nursing experience was also examined for relational growth. In this model, attachment avoidance remained negatively associated with relational growth, compassion satisfaction remained positively associated with relational growth, and attachment anxiety remained non-significant. Because age and years of nursing experience were highly collinear, this model was treated as supplementary rather than as the primary specification.

### 3.3 Attachment and professional quality of life

Hierarchical regression results examining compassion satisfaction, burnout, and secondary traumatic stress are presented in Table 3. Age was entered in the first step, followed by attachment anxiety and avoidance. The final model was significant, *R*^2^ = 0.051, adjusted *R*^2^ = 0.037, *F*(3, 204) = 3.66, *p* = 0.013. Attachment anxiety and avoidance explained an additional 3.6% of variance beyond age, Δ*R*^2^ = 0.036, Δ*F*(2, 204) = 3.87, *p* = 0.022. In the final model, greater attachment avoidance was associated with lower compassion satisfaction, *B* = −0.89, *β* = −0.19, *p* = 0.006. Neither age, *p* = 0.069, nor attachment anxiety, *p* = 0.686, was independently associated with compassion satisfaction. Although statistically significant, the final compassion satisfaction model explained a relatively modest proportion of variance (*R*^2^ = 0.051), indicating that attachment dimensions accounted for only a small share of individual differences in compassion satisfaction and that other personal, occupational, and organizational factors are likely important.

**Table 3:** Hierarchical regression analyses predicting professional quality of life outcomes.

Predictor	*B*	SE *B*	*β*	95% CI for *B*	*p*
Age	0.056	0.030	0.128	[−0.004, 0.115]	0.069
Attachment anxiety	0.125	0.309	0.029	[−0.484, 0.735]	0.686
Attachment avoidance	−0.893	0.321	−0.191	[−1.525, −0.260]	0.006
Model fit					
Block 1 *R*^2^	0.015				
Block 2 Δ*R*^2^	0.036				
Final *R*^2^	0.051				
Adjusted *R*^2^	0.037				
Final *F*(df)	3.66 (3, 204)				0.013

Predictor	*B*	SE *B*	*β*	95% CI for *B*	*p*
Age	0.021	0.028	0.044	[−0.033, 0.076]	0.440
High-intensity clinical setting	2.652	0.720	0.215	[1.231, 4.072]	<0.001
Attachment anxiety	1.527	0.291	0.309	[0.954, 2.101]	<0.001
Attachment avoidance	2.096	0.292	0.398	[1.520, 2.673]	<0.001
Model fit					
Block 1 *R*^2^	0.138				
Block 2 Δ*R*^2^	0.258				
Final *R*^2^	0.396				
Adjusted *R*^2^	0.384				
Final *F*(df)	33.28 (4, 203)				<0.001

Predictor	*B*	SE *B*	*β*	95% CI for *B*	*p*
Age	−0.106	0.032	−0.172	[−0.169, −0.044]	<0.001
High-intensity clinical setting	2.435	0.829	0.157	[0.802, 4.069]	0.004
Attachment anxiety	2.055	0.335	0.330	[1.395, 2.715]	<0.001
Attachment avoidance	2.992	0.336	0.450	[2.329, 3.655]	<0.001
Model fit					
Block 1 *R*^2^	0.182				
Block 2 Δ*R*^2^	0.316				
Final *R*^2^	0.498				
Adjusted *R*^2^	0.488				
Final *F*(df)	50.36 (4, 203)				<0.001

Unstandardized coefficients are reported with 95% confidence intervals. High-intensity clinical setting was coded 0 = no and 1 = yes. In the compassion satisfaction model, Block 1 included age and Block 2 added attachment anxiety and attachment avoidance. In the burnout and secondary traumatic stress models, Block 1 included age and high-intensity clinical setting; Block 2 added attachment anxiety and attachment avoidance.

Two additional hierarchical regressions examined burnout and secondary traumatic stress. In both models, age and high-intensity clinical setting were entered in the first step, followed by attachment anxiety and avoidance in the second step.

For burnout, age and high-intensity clinical setting explained 13.8% of the variance, *R*^2^ = 0.138, *F*(2, 205) = 16.47, *p* < 0.001. Attachment anxiety and avoidance explained an additional 25.8% of variance, Δ*R*^2^ = 0.258, Δ*F*(2, 203) = 43.30, *p* < 0.001. The final model explained 39.6% of the variance in burnout, adjusted *R*^2^ = 0.384, *F*(4, 203) = 33.28, *p* < 0.001. Working in at least one high-intensity clinical setting was associated with greater burnout, *B* = 2.65, *β* = 0.22, *p* < 0.001. Both attachment anxiety, *B* = 1.53, *β* = 0.31, *p* < 0.001, and attachment avoidance, *B* = 2.10, *β* = 0.40, *p* < 0.001, were independently associated with greater burnout. Age was not uniquely associated with burnout, *p* = 0.440.

For secondary traumatic stress, age and high-intensity clinical setting explained 18.2% of the variance, *R*^2^ = 0.182, *F*(2, 205) = 22.76, *p* < 0.001. Attachment anxiety and avoidance explained an additional 31.6% of variance, Δ*R*^2^ = 0.316, Δ*F*(2, 203) = 63.97, *p* < 0.001. The final model explained 49.8% of the variance in secondary traumatic stress, adjusted *R*^2^ = 0.488, *F*(4, 203) = 50.36, *p* < 0.001. Younger age, *B* = −0.11, *β* = −0.17, *p* < 0.001, and working in at least one high-intensity clinical setting, *B* = 2.44, *β* = 0.16, *p* = 0.004, were associated with greater secondary traumatic stress. Greater attachment anxiety, *B* = 2.06, *β* = 0.33, *p* < 0.001, and greater attachment avoidance, *B* = 2.99, *β* = 0.45, *p* < 0.001, were also independently associated with greater secondary traumatic stress.

No serious multicollinearity or case-influence concerns were identified in the primary models. Across final models, variance inflation factors ranged from 1.018 to 1.166, indicating no evidence of problematic multicollinearity. Inspection of residual and influence diagnostics did not suggest that individual observations unduly influenced the findings; in models for which influence statistics were retained, maximum Cook’s distance values ranged from 0.037 to 0.045.

## 4 Discussion

The present study examined associations among attachment insecurity, professional quality of life, and perceived posttraumatic growth among hospital-based nurses providing direct patient care. Overall, the findings supported the relevance of attachment-related processes to both negative and positive dimensions of nurses’ professional and psychological experiences. Attachment avoidance emerged as the most consistent correlate across outcomes: greater avoidance was associated with lower compassion satisfaction, greater burnout and secondary traumatic stress, and lower perceived posttraumatic growth reported by nurses, including growth in relating to others. Attachment anxiety was also associated with greater burnout and secondary traumatic stress but was not uniquely associated with compassion satisfaction or perceived posttraumatic growth after accounting for the other variables. In addition, compassion satisfaction was positively associated with both relational growth and total perceived posttraumatic growth.

Because the present study used a cross-sectional design, the associations discussed below should not be interpreted as evidence of temporal or causal relationships. Proposed explanations involving attachment-related emotion regulation and relational processes are therefore theoretical interpretations of the observed associations and require longitudinal or experimental investigation.

The findings regarding attachment avoidance are consistent with attachment theory and prior work on emotion regulation. Individuals higher in avoidance tend to rely on deactivating strategies, including emotional distancing and counterdependence, suppression of vulnerability, and reduced reliance on others during times of stress (Eilert and Buchheim, 2023; Mikulincer and Shaver, 2019). In nursing contexts, these strategies may be associated with lower support seeking, less open engagement with emotionally demanding experiences, and reduced interpersonal reward from caregiving work. This interpretation is consistent with prior research linking attachment insecurity with emotional exhaustion and caregiving-related difficulties among nurses (Golia et al., 2017). In the present study, avoidance was associated not only with higher burnout and secondary traumatic stress but also with lower compassion satisfaction, suggesting that emotional distancing may be relevant to both the burdens and rewards of nursing work.

Attachment avoidance was also independently associated with lower scores on the PTGI Relating to Others subscale and on overall perceived posttraumatic growth reported by nurses. This pattern is theoretically coherent, particularly for relational growth. The Relating to Others domain reflects perceived increases in emotional closeness, appreciation of support, willingness to express emotions, and investment in relationships. Because avoidant attachment is characterized by discomfort with dependence and interpersonal closeness, higher avoidance may be associated with fewer opportunities to process difficult experiences through supportive relationships or with greater difficulty perceiving relational change following adversity. Prior research has similarly suggested that attachment insecurity may be associated with lower posttraumatic growth, although the strength and direction of these associations may vary across attachment dimensions and contexts (Gleeson et al., 2021; Huh et al., 2020).

In contrast, attachment anxiety showed a more circumscribed pattern. Higher attachment anxiety was associated with greater burnout and secondary traumatic stress, but it was not uniquely associated with compassion satisfaction or perceived posttraumatic growth in the multivariable models. This finding may reflect the hyperactivating emotion-regulation strategies often associated with attachment anxiety. Individuals high in attachment anxiety may be especially sensitive to interpersonal threat, more likely to experience distress intensely, and more likely to have difficulty disengaging from emotionally demanding experiences. In a nursing context, these tendencies are theoretically consistent with greater emotional strain and vulnerability to secondary traumatic stress. However, heightened emotional engagement may not necessarily preclude nurses from experiencing professional meaning or perceived growth. The absence of an independent association between attachment anxiety and posttraumatic growth may indicate that its association with growth is more complex, context-dependent, or explained by other factors not assessed in the present study, such as social support, reflective coping, deliberate rumination, or organizational climate.

Attachment avoidance was consistently associated with lower compassion satisfaction, greater burnout and secondary traumatic stress, and lower levels of perceived posttraumatic growth reported by nurses. This finding indicates an association between experiencing one’s professional role as meaningful and rewarding and reporting more positive changes following challenging life experiences. The cross-sectional design does not permit conclusions regarding the direction of this association. Greater fulfillment from helping others and greater perceived growth may covary because of reciprocal processes or because both are associated with other factors not assessed in the present study. This pattern is consistent with research suggesting that compassion satisfaction may function as an important resource in nurses’ adaptation to work-related stress and in the relationship between secondary traumatic stress and posttraumatic growth (Zeng et al., 2023).

The findings also highlight the importance of occupational context. Working in at least one high-intensity clinical setting was associated with greater burnout and secondary traumatic stress. This result is consistent with research documenting the emotional demands of direct-care nursing and the heightened risk of compassion fatigue-related outcomes among nurses exposed to intense clinical situations (Jarrad and Hammad, 2020; Sinclair et al., 2017). These findings reinforce that nurses’ professional quality of life reflects both individual psychological differences and the environments in which they work. Attachment-related tendencies may shape how nurses regulate and interpret emotionally demanding experiences, but organizational conditions, clinical workload, exposure to patient suffering, and access to support are also likely to be important.

Younger age was associated with greater secondary traumatic stress and greater perceived posttraumatic growth. These associations should be interpreted cautiously because age was strongly correlated with years of nursing experience. Age may reflect a combination of developmental, cohort, and professional-experience differences rather than a single explanatory factor. The present findings do not establish whether younger nurses are more vulnerable to emotional strain, more likely to perceive growth, or differ in the types of challenging experiences they have encountered. Future longitudinal research should more directly examine the respective roles of age, professional tenure, clinical setting, and cumulative exposure to stressful events.

### 4.1 Clinical and organizational implications

These findings have implications for resilience-oriented approaches to supporting nurses who work in emotionally demanding clinical environments. Attachment avoidance was associated with lower compassion satisfaction, greater burnout and secondary traumatic stress, and lower perceived posttraumatic growth. Attachment anxiety was also associated with greater burnout and secondary traumatic stress. Although attachment orientations are not clinical labels and should not be used to screen or categorize nurses, they may help identify relational and emotion-regulation processes that contribute to professional distress and well-being.

The observed associations identify emotional awareness, self-compassion, reflective coping, and supportive interpersonal connection as potentially relevant processes for future intervention research. Existing intervention research among nurses provides preliminary support for mindfulness-, self-compassion-, and resilience-oriented approaches, with systematic reviews and meta-analyses reporting improvements in resilience and selected indicators of psychological well-being following such interventions (Biber, 2025; Han and Yeun, 2024; Liu et al., 2025). Compassion- and mindfulness-informed approaches may therefore be particularly relevant to these processes; however, their effectiveness for the specific attachment-related and professional quality-of-life outcomes examined in the present study cannot be inferred from our findings and requires direct evaluation in intervention studies. Future research could also examine how such approaches might be integrated with organizational strategies such as peer consultation, reflective practice groups, supervision, manageable workloads, and psychologically safe environments in which nurses can discuss difficult clinical experiences.

Importantly, the present findings do not establish that compassion-focused or mindfulness-based interventions would reduce burnout or secondary traumatic stress. Rather, they identify attachment-related emotion regulation, professional meaning, and relational connection as potentially relevant targets for future resilience-enhancing interventions.

### 4.2 Strengths and limitations

This study has several strengths. It examined both negative and positive dimensions of professional quality of life, included a focus on perceived posttraumatic growth rather than occupational distress alone, and assessed attachment anxiety and avoidance dimensionally. The sample was limited to nurses providing direct patient care in hospital settings, allowing the study to focus specifically on a population exposed to substantial emotional demands. In addition, internal consistency estimates for the study measures were good to excellent.

The findings should also be interpreted in light of several limitations. First, the cross-sectional design precludes causal conclusions. Attachment insecurity, professional quality of life, and perceived posttraumatic growth may influence one another over time, and longitudinal research is needed to clarify temporal relationships. Second, all primary variables were assessed using self-report measures within a single online survey session. As a result, some of the observed correlations and regression associations may partly reflect shared method variance, response styles, or contemporaneous affect rather than exclusively substantive relationships among the constructs. Future studies would benefit from incorporating multiple methods or sources of data, such as prospective assessments, organizational indicators, or supervisor- and peer-reported measures. The study also did not directly assess several organizational and workplace determinants that may influence professional quality of life, including staffing levels, workload, scheduling demands, organizational support, leadership climate, psychological safety, and frequency or intensity of exposure to patient suffering. Accordingly, the observed associations with attachment should not be interpreted as indicating that individual relational characteristics are more important than organizational or structural determinants of burnout and secondary traumatic stress. Future studies should examine attachment-related processes alongside organizational and structural predictors to clarify their relative and combined contributions to nurses’ occupational well-being. Third, the PTGI was not anchored to a specific nursing-related event or occupational experience. The findings therefore concern perceived posttraumatic growth among nurses rather than growth attributable specifically to nursing work. Fourth, the high-intensity clinical-setting indicator was a broad binary variable and may not have captured differences in the frequency, duration, or subjective impact of stressful clinical exposures. Future studies should assess clinical work intensity using graded and multidimensional indicators, including unit type, frequency and duration of exposure to acute or traumatic events, patient acuity, staffing ratios, workload, shift characteristics, cumulative exposure, and the perceived intensity or impact of occupational stress. Finally, participants were recruited through hospital networks using a nonprobability convenience-sampling approach rather than stratified or probability-based sampling across geographic regions or healthcare institutions. Accordingly, the sample should not be considered nationally representative of U.S. hospital nurses, and selection effects may limit the generalizability of the findings to nurses working in other settings, regions, or healthcare systems. In addition, because the number of nurses who received or viewed the recruitment invitation was not available, response rate could not be calculated and the extent of nonresponse or self-selection bias cannot be fully determined.

### 4.3 Future directions

Future research would benefit from longitudinal and multisite designs that assess nurses’ exposure to specific occupational stressors, organizational support, staffing conditions, social support, and coping processes. Studies could also examine whether compassion satisfaction, self-compassion, mindfulness, or supportive workplace relationships mediate associations between attachment insecurity and professional quality of life.

Intervention-based research could clarify whether resilience- or compassion-focused approaches improve professional quality of life among nurses with differing attachment-related patterns. For example, nurses higher in attachment avoidance may benefit from interventions that emphasize gradual emotional engagement, interpersonal safety, and accessible forms of peer support, whereas nurses higher in attachment anxiety may benefit from approaches that strengthen emotion regulation and reduce perseverative responses to stressful experiences. Measures of posttraumatic growth should also be anchored to clearly identified occupational stressors or clinical experiences to clarify whether and how difficult nursing experiences contribute to perceived changes in relationships, meaning, and personal functioning.

## 5 Conclusion

This study highlights the relevance of attachment insecurity to both the negative and positive dimensions of nurses’ professional experiences. Attachment avoidance was consistently associated with lower compassion satisfaction, greater burnout and secondary traumatic stress, and lower perceived posttraumatic growth reported by nurses. Attachment anxiety was associated primarily with burnout and secondary traumatic stress. Compassion satisfaction was positively associated with perceived relational and overall posttraumatic growth.

Together, these findings support a resilience-oriented approach to nurse well-being that considers occupational conditions alongside emotional and relational processes. Supporting nurses’ capacity for self-compassion, reflective coping, interpersonal connection, and meaningful engagement in their work may represent promising directions for future clinical and organizational interventions.

## Data Availability

The raw data supporting the conclusions of this article will be made available by the authors, without undue reservation.
